# Next-Generation Biomarkers and Artificial Intelligence in Colorectal Cancer: From Multi-Omic Data Integration to Clinical Application

**DOI:** 10.3390/cancers18183052

**Published:** 2026-09-20

**Authors:** Christina Loukopoulou, Ioannis Koliarakis, John Tsiaoussis

**Affiliations:** Department of Anatomy, School of Medicine, University of Crete, 71003 Heraklion, Greecetsiaoussis@uoc.gr (J.T.)

**Keywords:** colorectal cancer, artificial intelligence, machine learning, deep learning, multi-omics, liquid biopsy, circulating tumour DNA, radiomics, computational pathology, immunotherapy, precision medicine

## Abstract

Colorectal cancer (CRC) remains a leading cause of cancer-related mortality worldwide, although advances in molecular profiling and artificial intelligence (AI) are now reshaping precision oncology in ways that promise better patient outcomes. This review synthesises contemporary evidence on next-generation biomarkers and AI applications in CRC across multi-omic data integration, liquid biopsy, computational pathology, radiomics and clinical implementation. Several findings stand out. Integrated multi-omic approaches combining genomics, transcriptomics, proteomics, metabolomics and microbiomics outperform single-omic biomarkers for predicting prognosis and treatment response. Machine learning (ML) and deep learning (DL) models achieve clinical-grade performance for molecular biomarker prediction directly from routine histopathology, with a pooled area under the receiver operating characteristic curve (AUROC) of 0.94 for microsatellite instability (MSI) detection. Circulating tumour DNA (ctDNA) monitoring enables minimal residual disease (MRD) detection and real-time treatment guidance, postoperative ctDNA status separating a two-year recurrence-free survival of 91.1% from 50.4%; randomised evidence further shows that ctDNA-guided management can safely reduce adjuvant chemotherapy use in stage II colon cancer. Radiomics and pathomics extract prognostically significant quantitative features from imaging and histopathology, permitting non-invasive tumour characterisation, while multimodal models integrating clinical, genomic, imaging and pathological data support individualised treatment selection. MSI-high (MSI-H) status predicts exceptional immunotherapy benefit, carrying an overall survival hazard ratio of 0.35 against chemotherapy. Considerable difficulties nonetheless persist: harmonising data across institutions, generalising models to diverse populations, algorithmic bias and regulatory frameworks suited to clinical AI. Priorities for the coming years are prospective validation of AI-guided treatment algorithms, integration of spatial transcriptomics and single-cell approaches, federated learning to support multi-institutional collaboration without compromising privacy, and standardised protocols for biomarker testing and interpretation.

## Simple Summary

Doctors treating colorectal cancer increasingly rely on measurements taken from the tumour, from blood samples and from scans to decide who needs chemotherapy, who is likely to benefit from immunotherapy and who might safely avoid further surgery. Computer programs that learn patterns from data are now applied to all of these measurements, and some can recognise features in a tissue slide that previously required a separate laboratory test. This review gathers the evidence behind these tests and computer methods and asks one question of each: Is it strong enough to show that using the test actually helps patients, or only that the test makes accurate predictions? These are not the same thing. The methods reporting the most impressive accuracy are often those tested least rigorously. By contrast, the two approaches that have genuinely changed how patients are treated were established through carefully controlled trials: a blood test that detects cancer left behind after surgery and a tumour feature that identifies patients likely to respond to immunotherapy. We also show that several promising measurements have never reached the clinic, not because the underlying biology was wrong, but because laboratories cannot yet measure them consistently enough for results to be compared.

## 1. Introduction and Clinical Context

### 1.1. Colorectal Cancer Epidemiology and the Biomarker Revolution

Colorectal cancer represents the third most commonly diagnosed malignancy and the second leading cause of cancer-related mortality worldwide. Of an estimated 20 million new cancer cases documented globally in 2022, roughly a quarter arose in the gastrointestinal tract, with CRC being the single most prevalent gastrointestinal malignancy at 9.6% of all new diagnoses [1]. Its clinical management has changed considerably over the past decade under the influence of molecular diagnostics and precision medicine [2]. Despite available screening tools, a substantial proportion of cases are still diagnosed at advanced stages, where survival is markedly worse than with early detection [3]. Endoscopy remains the modality of choice for secondary prevention, since it permits diagnosis and treatment in a single session. Adherence to endoscopic protocols is nevertheless lower than intended, partly on account of the invasiveness of the procedure [1]. This is the practical case for developing non-invasive biomarkers alongside existing pathways, not as replacements for them. More than half of gastrointestinal malignancies are attributable to modifiable exposures such as diet, alcohol, smoking, obesity and physical inactivity [1]. Specific exogenous agents have also been examined for their carcinogenic contribution; the role of anabolic agents in colorectal carcinogenesis, for example, rests on weaker evidence than is often assumed [4]. Primary prevention has made limited headway against these exposures, which places the burden on earlier and more precise detection. The conceptual foundation for this work was laid decades ago. The adenoma–carcinoma sequence described by Fearon and Vogelstein set out a stepwise accumulation of genetic alterations through *APC*, *KRAS* and *TP53*, and it remains the organising model of colorectal carcinogenesis [5]. It built on the earlier demonstration that such alterations accumulate progressively across the stages of tumour development [6] and was later extended by work on hereditary syndromes that clarified the distinct mismatch repair pathway [7]. Early detection and surgical resection remain the cornerstones of management, but next-generation biomarkers combined with machine learning (ML) now offer scope for better patient stratification, treatment selection and outcome prediction. By integrating genomic data with conventional clinical variables, oncologists can identify actionable alterations and adapt treatment accordingly, moving beyond one-size-fits-all protocols [8].

The complexity of CRC biology, characterised by substantial inter- and intra-tumoural heterogeneity, necessitates comprehensive molecular profiling beyond single-gene assessment. Tissue biopsy-based testing, while clinically valuable, provides only a snapshot of tumour genomics at a single time point and location, potentially missing important clonal variants and dynamic changes during treatment. This limitation has prompted diagnostic approaches that draw on several biological data streams at once. ML prediction models applied to m6A RNA methylation regulators, for example, achieve validation AUROC values of 0.847, above which conventional approaches deliver [9]. Biomarker research has consequently shifted from univariate discovery toward integrated multi-omic platforms covering the genetic, epigenetic, transcriptomic, proteomic, metabolomic and microbial dimensions of the tumour [10,11].

### 1.2. From Single Biomarkers to Multi-Omic Integration

Traditional CRC biomarkers such as *KRAS*, *TP53*, *BRAF* and MSI have provided critical clinical guidance for decades, yet each operates within a limited biological context. *KRAS* mutations, present in approximately 40–50% of CRC cases, predict resistance to anti-epidermal growth factor receptor (EGFR) monoclonal antibodies and constitute a critical decision point for treatment selection [8]. *BRAF* V600E mutations, occurring in 7–10% of metastatic CRC (mCRC), indicate an aggressive disease phenotype with poor prognosis and specific therapeutic implications [12]. Mutations in *KRAS*, *NRAS* and *BRAF* are linked to anti-EGFR resistance, making their evaluation an essential component of precision diagnostics [13]. However, these binary categorisations fail to capture the full spectrum of tumour biology and cannot adequately predict treatment response in many patients. Comprehensive appraisals of the field have accordingly organised candidates by compartment (tissue-derived, blood-derived and faecal molecular markers) instead of by single gene, on the argument that diagnosis, prognosis and prediction pose distinct clinical questions and require distinct marker sets [14].

Advanced ML approaches have identified neutrophil extracellular trap (NET)-related gene signatures achieving an AUROC of 0.745–0.762 for predicting recurrence and chemotherapy response [15]. Hypoxia-related gene signatures combined with ML algorithms achieved an AUROC of 0.9663 for predicting cetuximab response [16]. A comprehensive analysis integrating DNA methylation, transcriptomics and immune infiltration identified IL-1β as both a diagnostic and prognostic biomarker with reported accuracy between 99% and 100% using ML approaches [17]. Taken together, these studies indicate that multi-dimensional integration can outperform single-gene assessment. The very high accuracies reported in retrospective, single-cohort analyses do, however, warrant caution until externally validated.

Contemporary evidence indicates that no single omic layer captures sufficient biological information for optimal CRC patient stratification. Integrated analysis of multiple omic data types improves prediction of treatment response, prognosis and drug resistance mechanisms compared with single-omic approaches [10,18]. A comprehensive integrative framework incorporating microbiota, tumour microenvironment and multi-omic data identified distinct CRC subtypes with differential survival outcomes and immunotherapy responses [19].

Multi-omic profiling is especially useful in CRC because tumour heterogeneity and therapeutic resistance arise through several mechanisms at once. ML analysis of ferroptosis and fatty acid metabolism produced an integrated signature (FeFAMscore) achieving a C-index of 0.689 in training cohorts and 0.648–0.720 in validation cohorts, outperforming existing models for predicting immunotherapy and chemotherapy response [20]. Integration of microbiota, metabolomic and imaging phenotype data has revealed associations between gut bacterial composition, systemic metabolite levels and immunotherapy response, highlighting how extra-tumoural factors influence treatment outcomes [19,21].

### 1.3. Artificial Intelligence in Precision Oncology

What artificial intelligence contributes to precision oncology is pattern recognition at a scale and dimensionality that manual analysis cannot reach: relationships between genomic alteration, tumour behaviour and treatment response that are present in the data but not apparent to inspection. ML now supplies much of the analytical capacity for extracting biological insight from complex, heterogeneous datasets in oncology. Supervised, unsupervised and deep learning approaches trained on diverse cancer datasets can recover non-linear relationships between molecular features and clinical outcomes that conventional statistical analysis leaves obscured, and they are increasingly applied to feature selection, dimensionality reduction and multi-modal integration [22,23]. Because these methods can process genomic alterations, gene expression, protein levels and metabolite concentrations together, they permit predictive biomarkers that reflect the biological complexity of individual tumours [18].

Recent architectural advances have driven much of this progress. Self-supervised pretraining, multimodal transformer architectures, graph neural networks, attention-based multiple-instance learning (MIL) and generative models now support generalised biomedical representation learning across radiology, digital pathology, genomics, transcriptomics, proteomics, metabolomics, epigenomics, spatial biology and clinical outcome data [22,24,25]. Explainable AI (XAI) techniques supply the transparency required for clinician confidence, regulatory review and trustworthy deployment: attention visualisation, saliency mapping, feature attribution, SHapley Additive exPlanations (SHAP), Gradient-weighted Class Activation Mapping (Grad-CAM) and uncertainty estimation [26,27].

The clinical utility of ML in oncology has been demonstrated across multiple CRC management domains. In diagnosis, models integrating clinical features, imaging and molecular data achieve high diagnostic accuracy for distinguishing CRC from benign conditions [28]. In prognosis, DL models trained on multi-modal datasets can outperform TNM staging in predicting disease-free and overall survival, identifying high-risk patients who might benefit from adjuvant intensification [29]. In treatment prediction, integrative frameworks analysing multi-omic data stratify patients by predicted immunotherapy response [30]. Ensemble ML approaches integrating genomics, transcriptomics, histopathology and clinical data have achieved, in conference abstract reports, an AUROC of approximately 0.80 for classifying immune checkpoint inhibitor (ICI) response in non-small cell lung cancer, with comparable performance now being reported in CRC [31,32].

### 1.4. Scope and Literature Search

**Figure 1:**
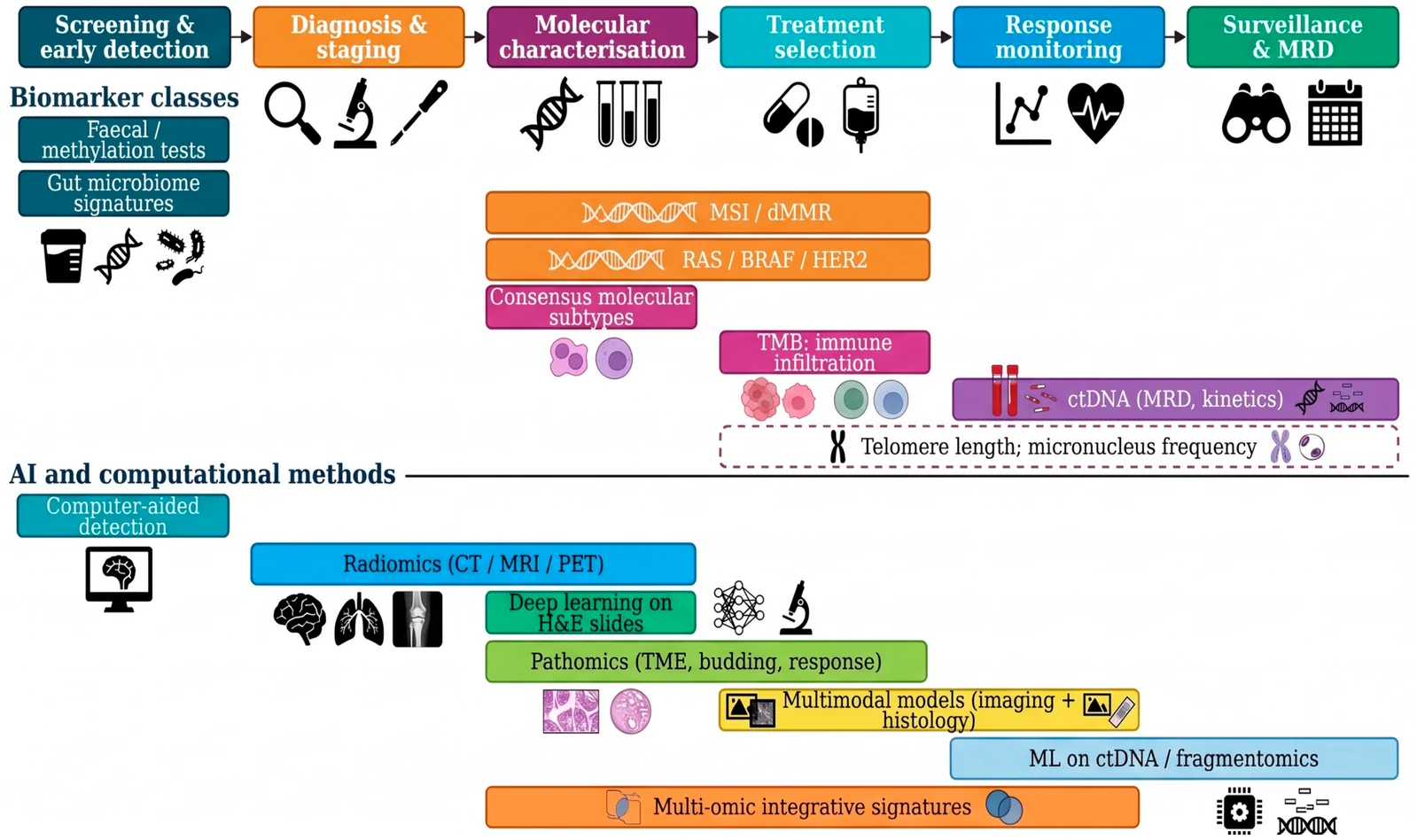
Biomarker classes and artificial intelligence methods mapped onto the colorectal cancer care pathway. Bars span the stages at which each approach is applied and are coloured by the pathway stage at which they are principally used; colour does not encode evidence. By strength of supporting evidence, faecal and methylation tests, MSI/dMMR, RAS/*BRAF*/*HER2* status, ctDNA and computer-aided detection are established in routine practice; consensus molecular subtypes, tumour mutational burden with immune infiltration, radiomics and deep learning applied to haematoxylin and eosin-stained slides have been externally validated but are not yet routine; gut microbiome signatures, pathomics, multimodal models, machine learning on ctDNA and multi-omic integrative signatures rest on retrospective evidence only; telomere length with micronucleus frequency, shown with a dashed outline, remains candidates because measurement is not standardised (Section 2.7). Approaches in routine use therefore cluster at molecular characterisation and minimal residual disease monitoring, whereas the densest activity, in multi-omic and multimodal modelling, is the least well evidenced.

This article is a narrative review rather than a systematic review, and no formal protocol was registered. PubMed/MEDLINE, Scopus and Web of Science were searched from database inception to February 2026, using combinations of the terms colorectal cancer, biomarker, multi-omics, liquid biopsy, circulating tumour DNA, minimal residual disease, digital pathology, radiomics, microbiome, microsatellite instability, artificial intelligence, machine learning and deep learning. Reference lists of retrieved articles and relevant reviews were screened for additional sources. Publications in English reporting original data, systematic reviews, meta-analyses or randomised trials in colorectal cancer were eligible. Priority was given to studies reporting external validation or prospective design, and, where several reports addressed the same question, to the largest or most recently published. Foundational papers were retained irrespective of date where they established a concept on which later work depends. Conference abstracts were included only where they represent the only available source for a specific finding and are identified as such in the text and in the reference list. Because selection was not exhaustive, the review should be read as a critical synthesis of the principal evidence rather than a complete enumeration of it. The scope of the review, and the relationship between the biomarker classes and computational methods it covers, are summarised in Figure 1.

## 2. Multi-Omic Biomarker Discovery and Machine Learning Integration

### 2.1. Genomic and Epigenomic Biomarkers with Clinical Implementation

Mutations in driver genes such as *APC*, *KRAS*, TP53 and BRAF identify therapeutic targets and form the cornerstone of molecular diagnostics in CRC [2]. Systematic characterisation came with The Cancer Genome Atlas analysis of colon and rectal tumours. It established that the two sites share a common pattern of genomic change, identified a hypermutated subgroup comprising roughly 16% of cases and defined the recurrently altered pathways (WNT, RAS-MAPK, PI3K, TGF-β and *TP53*) on which subsequent subtyping efforts, including CMS, were built [33]. RAS and *BRAF* mutation status, human epidermal growth factor receptor 2 (*HER2*) amplification and MSI/mismatch repair deficiency (dMMR) status are now standard components of therapeutic decision-making, particularly for anti-EGFR therapy selection [2].

*KRAS* mutations are among the most clinically consequential biomarkers in CRC, and the evidence establishing them as predictive rather than merely prognostic is unusually clean. A retrospective analysis of a randomised panitumumab trial showed progression-free survival benefit confined to the wild-type *KRAS* stratum (hazard ratio: 0.45) with no meaningful effect in mutant tumours (hazard ratio: 0.99) [34], and a parallel analysis of cetuximab in chemotherapy-refractory disease reached the same conclusion [35]. Extended RAS testing subsequently proved necessary. Mutations beyond *KRAS* exon 2, in *KRAS* exons 3 and 4 and *NRAS* exons 2–4, likewise predicted absence of benefit; patients with any RAS mutation fared worse when panitumumab was added to FOLFOX4 [36]. This progression from single-exon to extended RAS testing is an instructive precedent for the biomarkers discussed below, since the initial marker was correct but incompletely specified. Even so, roughly 40% of RAS wild-type tumours fail to respond, so additional biomarkers remain necessary for precise selection [8]. *KRAS* subtypes (G12C, G12V, G12D and G12S) respond differently to MEK and *KRAS*-selective inhibitors, which suggests that subtype-level categorisation improves treatment planning over a binary mutated/wild-type call.

*TP53* mutations, affecting approximately 50–70% of CRCs depending on stage and molecular subtype, associate with aggressive phenotype and generally predict poor chemotherapy response, yet their prognostic significance varies substantially with subtype and immune microenvironment context. Integrating *TP53* status with additional biomarkers improves prognostic accuracy; *TP53*-mutant tumours enriched for tumour-infiltrating lymphocytes can exhibit favourable immunotherapy responses despite otherwise poor prognostic features.

DNA methylation patterns represent an epigenetic layer complementary to somatic mutations. A CpG methylation signature (the CENSURE assay), reported to date only in conference abstract form, achieved an AUROC of 91.2% in training and 79.3% in testing cohorts for predicting postoperative recurrence in stage II/III CRC [37]. This signature, integrating six CpG methylation sites with carcinoembryonic antigen (CEA), identified an additional 46.5% of recurrence cases that CEA alone missed. Blood-based epigenetic biomarkers are especially promising for early detection, since methylation changes are detectable in plasma early during carcinogenesis [38].

### 2.2. Transcriptomic Profiling and Consensus Molecular Subtypes

Transcriptomic profiling enables classification of CRC into clinically meaningful subtypes and informs prediction of drug sensitivity. The consensus molecular subtype (CMS) classification arose from an international consortium formed specifically to resolve inconsistencies between six competing gene expression-based classifications, which were shown to converge on four subtypes [39]. In the consortium’s cohort, these were CMS1 (MSI-immune, 14%), hypermutated and microsatellite unstable with strong immune activation, and CMS2 (canonical, 37%), epithelial with marked WNT and *MYC* signalling. The remaining two were CMS3 (metabolic, 13%), epithelial with evident metabolic dysregulation and frequent *KRAS* mutations, and CMS4 (mesenchymal, 23%), with prominent TGF-β activation, stromal infiltration and the worst prognosis [39,40,41]. Reported subtype proportions vary by a few percentage points across later cohorts depending on platform and case mix.

The clinical utility of CMS extends beyond prognosis to treatment response prediction. CMS is prognostic in both adjuvant and metastatic settings, with CMS2 linked to the best survival and CMS4 linked to the worst survival [40]. Molecular analysis of the AGITG MAX trial established the prognostic impact of CMS and its predictive effect for bevacizumab benefit in mCRC [42]. Broadly, CMS1 responds favourably to immunotherapy, CMS2/3 responds favourably to bevacizumab-containing regimens, and CMS4 may benefit from irinotecan-based therapy [40]. Image-based CMS classifiers using deep learning (imCMS) have enabled cost-effective molecular subtyping directly from routine histopathology slides [43], with predicted CMS1 cases showing significantly higher likelihood of pathological complete response to neoadjuvant chemoradiotherapy [44]. The fact that a transcriptomic classifier can be recovered from morphology alone has practical consequences, since RNA-based CMS assignment demands tissue quality and expenditure that routine practice often cannot supply.

### 2.3. Proteomic, Metabolomic and Microbiome-Derived Signatures

Proteomics resolves differences in functional proteins relevant to therapy, while metabolomics identifies characteristic metabolites reflecting tumour metabolic reprogramming. Integration of metabolomic and transcriptomic data has enabled construction of prognostic models with good discriminative value, with differential analysis identifying metabolites significantly altered between normal and tumour tissue and enrichment analysis revealing the associated signalling pathways [45].

Blood-based biomarker discovery using proteomics and metabolomics has gained substantial attention because of accessibility, reproducibility and potential for early detection [46]. Non-coding RNAs, including microRNAs, circular RNAs and PIWI-interacting RNAs, have joined disease-specific proteins and metabolic profiles as candidates for non-invasive detection, with particular potential for identifying adenomas before malignant transformation [46].

The gut microbiome has emerged as a promising source of non-invasive biomarkers, with faecal metagenomic studies consistently identifying a core CRC-associated signature enriched for oral-typical, biofilm-forming species [47]. The fact that the discriminating taxa are characteristically oral, not colonic, is no accident. The oral cavity and colon are both densely colonised and anatomically connected. Ectopic colonisation of the gut by oral bacteria has been proposed as a mechanism linking periodontal dysbiosis to colorectal carcinogenesis, with translocated species establishing themselves in the tumour microenvironment and sustaining local inflammation [48]. This bears directly on assay design: It suggests the oral cavity as an alternative and more accessible sampling compartment than stool, and it implies that a faecal signature dominated by oral taxa reports on a translocation process, not on resident colonic flora. *Fusobacterium nucleatum*, *Parvimonas micra*, *Peptostreptococcus stomatis* and *Bacteroides fragilis* have been repeatedly identified as key taxa associated with CRC development and progression [47].

Specific bacterial signatures including *F. nucleatum* and *P. micra* correlate with stage and metastasis, supporting their diagnostic potential [49]. Combining metagenomic with metabolomic data enhances diagnostic accuracy substantially over either alone [3]. A four-genus cluster of *Fusobacterium*, *Parvimonas*, *Bacteroides* and *Faecalibacterium* has been proposed as the basis for a non-invasive faecal test for early CRC diagnosis [50]. Multi-omic profiling has further revealed associations between the gut microbiome, host genome and host transcriptome, situating microbial signals within the tumour’s wider molecular context instead of treating them as an isolated data layer [21].

One practical caveat applies to any longitudinal use of these markers. The perioperative period itself substantially alters the intestinal microbiota, through mechanical bowel preparation, antibiotic prophylaxis, anaesthesia and the resection. These alterations bear on anastomotic healing and postoperative outcome [51]. A microbiome-derived signature measured before and after surgery is therefore not measuring the same baseline, which constrains the use of such markers for postoperative monitoring in the way ctDNA is used (Section 3.2) and makes them better suited to pre-treatment diagnosis and risk stratification than to serial surveillance.

A mechanistic link between the microbiome and genomic instability runs through oxidative stress. Oxidative stress arises from an imbalance between reactive oxygen and nitrogen species (ROS/RNS) and antioxidant defences. The gut microbiota modulates this balance through metabolite production, regulation of antioxidant enzymes and maintenance of intestinal homeostasis, while oxidative stress itself can promote dysbiosis. Microbial metabolites such as the short-chain fatty acids acetate, propionate and butyrate exert antioxidant effects. Conversely, dysbiosis may increase ROS generation and impair the intestinal barrier, facilitating the translocation of harmful molecules and antigens that further enhance oxidative stress and inflammation. ROS-mediated activation of NF-κB promotes pro-inflammatory cytokine expression and anti-apoptotic signalling. In CRC, excessive ROS contributes to tumour initiation and progression by supporting epithelial proliferation, angiogenesis, epithelial–mesenchymal transition and resistance to apoptosis. Both pathogenic and potentially protective bacteria can influence redox homeostasis; however, direct comparisons of their ROS-generating capacity in standardised CRC models remain limited [1,52]. The consequence for genomic stability is that reactive oxygen species oxidise guanine to 8-oxo-guanine and generate strand breaks, and because telomeric DNA is guanine-rich, it is disproportionately susceptible to this damage and is repaired less efficiently than the rest of the genome. Oxidative damage therefore accelerates telomere attrition (Section 2.7), which places the microbiome upstream of one of the substrates of chromosomal instability rather than merely correlated with disease status. Integrated microbial, metabolomic and telomere measurement is well placed to test that hypothesis.

### 2.4. Machine Learning Applications to Microbiome Data

Taxonomic abundance profiles of the kind described in Section 2.3 are high-dimensional, sparse and compositional. This makes them poorly suited to the univariate statistics that first identified them, and a natural target for the computational methods discussed throughout this review. ML provides the computational framework for extracting diagnostically relevant patterns from them. Recent models based on stool microbiome data have achieved AUROC values ranging from 0.61 to 0.98 in internal validation and 0.70 to 0.87 in external validation cohorts [53,54,55,56,57,58,59,60,61,62,63,64]. Random forest (RF) has been particularly widely applied, with reported AUROCs of 0.699–0.926 [53,55,56,58,60,62,63,64], whereas XGBoost-based approaches have achieved AUROCs of 0.90–0.98 [57,61]. These ensemble algorithms appear well suited to the dimensionality and complexity of microbiome data, although performance depends strongly on preprocessing, feature selection and validation strategy.

Integration of microbial information with additional omic and clinical variables may further improve discrimination. In early-onset CRC, a multi-omic ML approach incorporating metabolomic information achieved an AUROC of 0.98, compared with 0.61 for a microbiome-only model, highlighting the potential value of combining complementary biological signals [54]. Functional microbiome features may also be informative; ML analysis of microbial enzymes identified CRC-associated glycosidases, CoA-transferases and other enzymatic activities linked to organisms including *Escherichia coli*, *Klebsiella pneumoniae* and *Clostridioides difficile* [58]. More broadly, recent approaches have integrated metabolites, microbial enzymes, demographic information and clinical variables alongside microbiome profiles to enhance CRC classification [54,58].

ML models can also identify simultaneously the microbial features contributing to classification. Recurrently enriched CRC-associated taxa include *Porphyromonas*, *Peptostreptococcus*, *Fusobacterium*, *Parvimonas micra*, *Gemella morbillorum*, *Bacteroides fragilis* and *Streptococcus*, whereas butyrate-producing taxa such as *Faecalibacterium*, *Eubacterium*, *Roseburia intestinalis* and Lachnospiraceae are frequently depleted [53,54,55,56,57,58,59,60,61,62,63,64]. The overlap with the taxa reported by the differential abundance studies of Section 2.3 is informative rather than redundant for two reasons. Concordance at the genus level does not imply concordance at the species level, nor the converse, since biodiversity within a genus varies considerably; the methodologies differ, so recovery of the same taxa by feature-ranking approaches constitutes independent validation of the original ecological findings. More specific signatures may capture clinically relevant heterogeneity. *Parasutterella* and *Ruminococcaceae UCG-002* were associated with early-onset CRC [54], *Atopobium* and *Haemophilus* were associated with adenomas [60], and *Hungatella hathewayi* and *Methanobrevibacter smithii* were associated with stage IV/metastatic CRC [64]. Strain-level analysis further identified several *F. nucleatum* subspecies enriched in CRC [64].

Explainable AI approaches, particularly SHAP-based feature attribution, have been applied to connect model predictions with individual microbial taxa and pathways, thereby reducing the “black-box” nature of ML-based diagnostics [54,55,56,57,58,60]. The constraints on clinical translation are, however, the same ones encountered throughout this review: predominantly retrospective case-control designs, heterogeneous sequencing and bioinformatic pipelines, inconsistent reporting of diagnostic metrics and limited external validation [53,54,55,56,57,58,59,60,61,62,63,64]. Prospective multicentre evaluation in asymptomatic screening populations, standardised workflows and direct comparison with established stool-based screening methods are therefore essential before microbiome-AI models can be incorporated into routine CRC screening.

### 2.5. Machine Learning-Derived Signatures

A caution applies to reading these figures as a series. The algorithms differ less than the accompanying performance values suggest: random forests, gradient-boosted trees such as XGBoost and LightGBM, and penalised regression all perform supervised feature selection over high-dimensional expression matrices, and their relative ranking within any one study reflects cohort size, class balance and hyperparameter tuning at least as much as biology. What distinguishes these signatures is therefore not the learner but the biological axis each interrogates. Neutrophil extracellular traps, hypoxia, ferroptosis and fatty acid metabolism, neddylation and mitochondrial function are each proxies for a distinct component of the tumour microenvironment or of the cellular stress response, and the recurrence of prognostic signal across axes this different suggests that the models are recovering a shared underlying phenotype, broadly the balance between immune engagement and stromal or metabolic adaptation, rather than independent mechanisms. Read this way, the consistency of performance across unrelated gene sets is itself the finding, and it argues for integrative modelling rather than for any individual signature.

Recent analyses have employed ML to systematically discover prognostic signatures across biological modalities. A neddylation-related gene signature developed using ten ML algorithms achieved AUROCs of 0.979, 0.989 and 0.996 for one-, two- and three-year overall survival prediction, respectively [65]. A mitochondrial-related gene prognostic model developed using 101 combinations of ML algorithms identified high-risk patients with significantly shorter overall survival and diminished immunotherapy efficacy [66]. As with other retrospective signature studies, these discrimination estimates are derived largely from public expression datasets and require prospective confirmation.

Multi-omic ML integration has identified *CD151* as a key biomarker in metabolic syndrome-related early-onset CRC, with expression correlating with stromal remodelling and chemoresistance [67]. *SULF1*, *CXCL8* and *PBLD* expression patterns identified by ML achieved an AUROC above 0.9 for distinguishing CRC from normal controls [68]. A study combining single-cell RNA sequencing with ML identified *PMAIP1* as a regulatory hub linking apoptosis to immune microenvironment remodelling. *PMAIP1* expression served as an independent prognostic predictor while paradoxically recruiting CD8+ effector T cells and upregulating immune checkpoint molecules [69], an illustration of how ML can surface the biological paradoxes that underlie treatment resistance. A clinical validation protocol for an ML-derived biomarker signature predicting response to cytotoxic chemotherapy with or without targeted therapy in mCRC is now underway, marking the transition of this class of signature from discovery to prospective evaluation [70].

### 2.6. Immune-Related Biomarkers and Molecular Subtypes

Immune and stromal classification of CRC is associated with molecular subtype and is directly relevant to precision immunotherapy [41]. Immune subtyping of lymph node metastasis-negative CRC using ML identified two immune subtypes with distinct immune scores and ICI responsiveness: subtype C1 showed high immune scores and predicted responsiveness, while subtype C2 demonstrated favourable prognosis and early-stage characteristics [71].

That immune infiltration carries prognostic weight in CRC was first established by Galon and colleagues, who showed that the type, density and location of immune cells within colorectal tumours predicted clinical outcome more reliably than conventional histopathological staging [72]. The Immunoscore formalised this observation as quantitative assessment of CD3+ and CD8+ T cell densities at the tumour core and invasive margin. It was validated in an international consortium study of 2681 evaluable stage I–III colon cancer patients, across training, internal validation and external validation sets. In that study, it provided a reliable estimate of recurrence risk and supported adding an immune component to TNM classification [73]. Integrating Immunoscore with molecular features has produced prognostic nomograms with improved discrimination, and ML analysis of the tumour microenvironment has extended this approach to a wider set of immune and stromal features [29].

### 2.7. Telomere Biology and Chromosomal Instability: A Case Study in Translational Failure

The preceding sections describe biomarkers whose reported performance is high but whose clinical adoption is limited. Telomere biology is the longest-running instance of that pattern and shows why measurement rather than biology is usually the binding constraint.

The underlying observation is old and secure. Telomeres shorten with successive division, and telomerase, comprising the reverse transcriptase subunit TERT and the RNA template TERC, counteracts this attrition [1]. A 1994 assay detected telomerase activity in 90 of 101 tumour biopsies across twelve cancer types and in none of 50 normal tissues [74]; current estimates place reactivation in 85–90% of human tumours [1,75]. In colorectal tissue, the signal is consistent: activity has been reported in 81% of cancers against 22% of polyps and in 90% of tumours with none in normal colon [1,76]. However, the population-level picture is equivocal. A meta-analysis of 23,379 cases and 68,792 controls across 51 studies found an association between telomere length and gastrointestinal cancers but none with overall cancer risk [1]. A dependable tissue-level signal alongside an inconsistent circulating one is the signature of an assay problem rather than a biological one.

Prospective measurement confirms this reading. Micronucleus frequency and telomerase activity were assessed together in peripheral blood lymphocytes across treatment in metastatic and locally advanced disease [77], after earlier work showed that treatment itself modulates the micronucleus measurement [78]. A fall in micronucleus frequency of less than 29% between mid-treatment and baseline distinguished progressive disease with a 36% sensitivity and an 87.0% specificity, whereas telomerase showed only a non-significant trend (*p* = 0.07) [77]. The first is a rule-in measure, the inverse of the profile that gives ctDNA-based minimal residual disease detection its value (Section 3.2). The second demonstrates the confound directly: measured in lymphocytes, which are themselves telomerase-positive when activated, the assay cannot separate tumour signal from background. The absence of reliable hTERT antibodies compounds this by precluding routine immunohistochemistry [1].

The problem is one of fractionation rather than of colorectal biology. EpCAM-dependent platforms detect circulating tumour cells in only around 30% of ovarian cancer patients, whereas marker-independent enrichment by imaging flow cytometry or inertial microfluidic capture recovers substantially more [79,80]; a systematic review of liquid biomarkers in endometrial cancer reached the conclusion that recurs throughout this literature, that is, single circulating markers lack standalone accuracy and that harmonised pre-analytical protocols are prerequisites for adoption [81]. What a blood-based assay detects is therefore determined substantially by how the blood was fractionated, and standardising such measurement is shared infrastructure across tumour types rather than a disease-specific problem (Section 3.3 and Section 4.1). Thirty years of secure biology has not sufficed here, and there is no reason to expect the ML signatures catalogued above to clear a bar that telomerase has not.

## 3. Liquid Biopsy: Non-Invasive Molecular Monitoring and Precision Diagnostics

### 3.1. Circulating Tumour DNA: Technical Advances

Circulating tumour DNA detected in peripheral blood plasma enables non-invasive assessment of tumour genomics. ctDNA originates from primary tumours, metastatic lesions and circulating tumour cells, providing a dynamic reflection of tumour genome evolution during therapy [82]. CRC is particularly suited to a liquid biopsy-based approach given the considerable shedding of circulating tumour fragments [8]. The clinical case for ctDNA rests on two early demonstrations. Serial measurement of circulating mutant DNA was shown to track tumour dynamics in colorectal cancer patients through surgery and chemotherapy, with postoperative persistence predicting recurrence [83]. Detection was then characterised across 640 patients and multiple tumour types. ctDNA proved detectable in more than 75% of advanced pancreatic, ovarian, colorectal and several other cancers, but in under 50% of primary brain, renal, prostate and thyroid tumours; for clinically relevant *KRAS* mutations in metastatic CRC, sensitivity reached 87.2% and specificity reached 99.2% [84]. The tumour-type dependence explains why CRC has remained among the most tractable settings for liquid biopsy. Ultra-sensitive next-generation sequencing (NGS) platforms now achieve detection limits in the range of 0.01–0.1% allele frequency, enabling identification of rare variants present in a small fraction of circulating DNA molecules.

Two assay design philosophies dominate. Tumour-informed approaches identify patient-specific mutations through tissue sequencing and then track them in plasma over time, yielding high specificity; tumour-agnostic (plasma-only) approaches require no tissue and are logistically simpler but must distinguish tumour signal from clonal haematopoiesis and germline variation. The relative merits of each have been systematically compared, and the optimal strategy remains an active question that depends on stage, shedding and clinical purpose [85]. A retrospective analysis of 49 CRC patients, reported in abstract form, detected postoperative ctDNA in 75% of baseline samples, predominantly involving *TP53*, *APC*, *KRAS* and *PIK3CA* mutations; 75% of ctDNA-positive patients eventually relapsed compared with 13.6% of ctDNA-negative patients [86].

ctDNA variant allele frequency (VAF) provides prognostic information complementary to mutation presence or absence. Baseline ctDNA VAF predicts treatment response and survival in mCRC, with higher baseline burden associated with worse chemotherapy outcomes. The kinetics of ctDNA during treatment carry even greater prognostic significance: complete ctDNA clearance early in first-line therapy predicts improved progression-free and overall survival, while persistent or rising ctDNA indicates need for therapeutic modification [87,88].

### 3.2. Minimal Residual Disease Detection and Prospective Validation

Accurate identification of MRD after curative-intent resection remains an unmet need. ctDNA now serves as a prognostic and predictive biomarker across MRD detection, early recurrence monitoring, molecular profiling and prediction of therapeutic response [8,88].

The UK TRACC part B study, one of the largest resected CRC cohorts with tissue-free MRD detection, demonstrated a two-year recurrence-free survival of 91.1% in patients without detectable postoperative ctDNA versus 50.4% in those with detectable ctDNA (hazard ratio: 6.5). The landmark negative predictive value was 91.2%, and the median lead time from ctDNA detection to radiological recurrence was 7.3 months [89].

A systematic review and network meta-analysis, available as a conference abstract, found both preoperative and postoperative ctDNA detection associated with higher progression risk, with pooled hazard ratios of 5.23 (95% CI: 2.10–13.00) and 7.95 (95% CI: 5.30–11.91), respectively. Testing after completion of adjuvant therapy proved most effective for identifying high-risk patients [90]. In stage II disease specifically, a systematic review and meta-analysis found postoperative ctDNA positivity significantly increased recurrence risk with a pooled risk ratio of 3.66 [91]. Real-world evidence from large prospective cohorts confirmed that MRD positivity is associated with inferior disease-free survival; among MRD-positive patients, transient ctDNA clearance carried significantly worse outcomes than sustained clearance, while ctDNA-negative patients receiving adjuvant chemotherapy showed no significant benefit versus observation [92].

In stage III CRC, serial ctDNA analysis has been used to assess adjuvant therapy efficacy and characterise the clinical behaviour of recurrences, extending the role of ctDNA beyond simple MRD detection [93]. A plasma-only MRD assay in metastatic CRC following curative-intent treatment likewise predicted recurrence with strong discrimination [94]. Serial ctDNA surveillance detected recurrence with a median lead time approaching ten months compared with standard computed tomography [95]. In neoadjuvant settings, ctDNA persistence correlates with histopathological residual disease and predicts recurrence risk despite clinical complete response; in the COPEC trial subset analysis, ctDNA positivity rates differed significantly between good and poor responders at multiple time points [96].

Randomised evidence, rather than observational, settles the question. The DYNAMIC trial assigned patients with resected stage II colon cancer to ctDNA-guided or standard clinicopathological management and found the ctDNA-guided approach non-inferior for two-year recurrence-free survival (93.5% versus 92.4%) while nearly halving adjuvant chemotherapy use (15% versus 28%) [97]. Five-year outcomes confirmed durability: the recurrence-free survival was 88% versus 87% and the overall survival was 93.8% versus 93.3%, with ctDNA clearance achieved in 87.5% of treated ctDNA-positive patients and a higher-than-median postoperative mutant molecule concentration associated with substantially worse five-year recurrence-free survival (HR: 10.62; *p* = 0.005) [98]. No other result reviewed here demonstrates so clearly that a molecular biomarker can safely de-escalate treatment and not merely stratify risk. CIRCULATE, COBRA and ACT3 continue to test tumour-informed against tumour-agnostic platforms at other stages [8].

### 3.3. Methylation-Based Assays, Multi-Component Biopsies and Real-World Implementation

Beyond variant analysis, methylation-based approaches offer complementary information. A methylation-specific droplet digital polymerase chain reaction (PCR) multiplex assay demonstrated a 96.7% specificity with a 64.4% sensitivity in localised tumours and 89.2% in metastatic CRC, with ctDNA dynamics significantly associated with progression-free and overall survival [99]. A CRC-specific methylation biomarker-based digital PCR assay found that ctDNA positivity at one month or later after curative-intent surgery was associated with significantly worse recurrence-free survival [100]. Combining genomic and methylation signals in a single tissue-free assay, as in TRACC part B, improves MRD sensitivity over either signal alone [89].

Circulating tumour RNA provides complementary information, particularly regarding gene fusions and expressed mutations not captured by DNA-only panels. Extracellular vesicles arising from tumour and stromal cells, exosomes and microvesicles among them, encapsulate proteins, lipids and nucleic acids that carry biomarker information [82,101]. Extracellular vesicle isolation combined with AI-based analysis of blood biomarker combinations has enhanced early CRC detection beyond conventional markers such as CEA and CA19-9 [87].

Real-world genomic databases demonstrate that ctDNA-detected MSI-H status predicts robust immunotherapy response across pan-cancer cohorts. ctDNA testing identified MSI-H status in approximately 1.4% of advanced solid tumours, and patients receiving immunotherapy showed significantly prolonged progression-free survival compared with non-immunotherapy treatments, validating liquid biopsy-based biomarker detection for enabling precision therapy [102]. Comprehensive ctDNA NGS has also been used to characterise the real-world genomic landscape of gastrointestinal cancers across Asia and the Middle East, addressing the geographical narrowness of much existing genomic evidence [103].

Discordance between ctDNA kinetics and radiological response is common and clinically meaningful, and molecular assessment can identify residual disease that imaging does not resolve; the converse problem, in which imaging underestimates response, is addressed in Section 6.3.

Integration of ctDNA-guided treatment algorithms into practice could optimise therapeutic strategies and reduce unnecessary intervention, but challenges remain in assay standardisation, sensitivity for low-shedding tumours, regulatory approval and cost-effectiveness [104,105,106]. Standardisation of detection methods and large-scale validation are needed before ctDNA becomes routine [91,107].

### 3.4. AI-Enhanced Liquid Biopsy Analysis

The integration of AI with multi-omic approaches has enhanced the analytical power of ctDNA assays. ctDNA-based liquid biopsy enables dynamic, real-time tracking of tumour evolution and therapeutic resistance, with ML-enhanced algorithms improving signal detection for earlier and more precise identification of actionable mutations and MRD [108]. Advances in NGS and digital PCR, combined with AI-based analysis and multi-omic integration, have improved the clinical viability of ctDNA testing [107,108].

Nowhere is the case for ML clearer than in fragmentomics. Cell-free DNA fragmentation is non-random: profiles from healthy individuals reflect the nucleosomal patterning of white blood cells, while patients with cancer show altered genome-wide fragmentation. Applying an ML model to these features across 236 patients with breast, colorectal, lung, ovarian, pancreatic, gastric or bile duct cancer and 245 healthy individuals gave detection sensitivities ranging from 57% to over 99% across the seven cancer types at a 98% specificity, with an overall AUROC of 0.94 [109]. Distributed across millions of fragment-level measurements, the signal cannot be recovered by any single-feature test. ctDNA variant calling differs in this respect, since the biomarker remains interpretable without a model. Fragmentomics is also largely mutation-agnostic, which makes it complementary to mutation-based ctDNA analysis and not a substitute for it.

Comprehensive reviews describe the analytical stack now applied across the CRC liquid biopsy spectrum: ensemble classifiers for recurrence prediction, convolutional networks for fragmentomics and circulating tumour cell image analysis, recurrent architectures for longitudinal ctDNA trajectories, and large language models for interpreting genomic reports. The same reviews set out the corresponding failure modes, which are dataset imbalance favouring the majority class, retrospective single-institution training sets, pre-analytical batch effects that degrade cross-site performance and an absence of prospective evidence that AI-guided liquid biopsy decisions improve outcomes and not merely biomarker metrics [110].

## 4. Computational Pathology and Radiomics

### 4.1. Radiomics: Principles and Prognostic Applications

Radiomics encompasses the systematic extraction of quantitative features from medical images acquired by computed tomography (CT), magnetic resonance imaging (MRI), positron emission tomography (PET) and other modalities. Where visual radiological assessment depends on subjective interpretation of obvious tumour characteristics, radiomics applies mathematical algorithms to identify features that reflect tumour structure, internal heterogeneity and microenvironmental properties [111]. Extracted features fall into several categories: morphological characteristics (shape, size, surface texture); first-order intensity statistics capturing voxel value distributions; texture features revealing fine-scale patterns of variation; and higher-order measures of structural complexity.

The premise that such features carry biological and prognostic information was established across independent lung and head-and-neck cohorts, where a large panel of quantitative image descriptors was shown to capture intratumoural heterogeneity and to associate with underlying gene expression patterns and survival [112]. Multimodal radiomics integrating CT, MRI and PET enables multidimensional characterisation of tumour morphology and underlying biology, providing an expanded basis for precision-oriented decision-making [111]. Multi-regional approaches have demonstrated particular value in CRC, since intratumoural and peritumoural regions exhibit distinct biological characteristics with separate prognostic significance. A systematic review and meta-analysis of radiomics for preoperative prediction of lymph node metastasis established both the promise of the approach and the methodological variability across studies [113].

Development of radiomic prognostic models requires careful attention to measurement reliability. Segmentation methods (manual, semi-automated or fully automated), image preprocessing settings and analysis software all influence extracted features, and the lack of standardisation across acquisition and segmentation workflows remains a principal obstacle to generalisability, alongside heterogeneity in datasets and validation strategies [111,113]. Reproducibility evaluation through test–retest imaging or inter-observer variability assessment is essential for validating feature stability before clinical use.

An ML model based on MRI radiomic features achieved AUROCs of 0.758–0.835 for predicting expression of p53, synaptophysin and HER2 and the presence of perineural and vascular invasion in CRC [114]. FDG PET-based radiomics identified features associated with tumour mutational burden (TMB), with a k-nearest neighbours model achieving an AUROC of 0.791 for predicting high TMB status [115].

### 4.2. Deep Learning for Image Analysis and Segmentation

Automated DL-based image analysis systems have become increasingly prevalent for medical image interpretation, achieving high accuracy in polyp detection, tumour identification and tissue characterisation. Computer-aided detection (CADe) during colonoscopy is the application with the strongest randomised evidence base anywhere in this review. A randomised trial of real-time CADe reported an adenoma detection rate of 54.8% with the system against 40.4% without it. The gain held across polyp size categories and endoscopist experience levels [116]; an earlier randomised study has shown a comparable increase in adenoma detection, driven principally by additional small adenomas [117]. Since the adenoma detection rate correlates inversely with post-colonoscopy interval cancer, this is one of the few AI applications in CRC where the causal chain to patient benefit is short and well characterised. Whether the additional small lesions detected translate into mortality reduction remains the open question.

Encoder–decoder segmentation architectures with skip connections have become standard for automated tumour region definition, enabling precise boundary identification while preserving fine anatomical detail. Attention mechanisms further improve performance by adaptively weighting informative image regions while suppressing background noise. Three-dimensional approaches examining volumetric data outperform two-dimensional slice-by-slice methods by capturing tumour geometry and spatial relationships. Transfer learning from models pretrained on large general imaging corpora substantially improves accuracy in clinical applications where domain-specific training data remain limited [23].

### 4.3. Deep Learning Prediction of Molecular Biomarkers from Histopathology

The fact that deep learning can infer microsatellite instability from routine haematoxylin and eosin-stained slides was first demonstrated in gastrointestinal cancer in 2019 [118], and the approach has since matured into one of the most developed applications of AI in CRC, now reaching clinical-grade performance [24]. A systematic meta-analysis including 30 original studies found a pooled specificity of 0.86, a sensitivity of 0.90 and an area under the summary receiver operating characteristic curve of 0.94 for pathology slide-based DL detection of MSI [119].

Self-supervised, attention-based MIL architectures consistently outperform earlier supervised approaches while offering explainable visualisation of indicative regions and morphologies [24]. Attention-based MIL identifies clinically meaningful regions within large whole-slide images without pixel-level annotation, which makes training on routinely archived slides feasible at scale. Prediction of MSI and *BRAF* mutation has reached clinical-grade performance, whereas prediction of *PIK3CA*, *KRAS* and *NRAS* status remains clinically insufficient [24]. Weakly supervised approaches have been applied specifically to *KRAS*, *NRAS*, *BRAF* and *HER2* status prediction, with performance consistent with this pattern [13]. Deepath-MSI, a feature-based MIL model designed for sensitive and specific MSI prediction, achieved an AUROC of 0.98 in test sets, with a 92% specificity and a 92% overall accuracy at a predetermined 95% sensitivity threshold [120].

Cross-tumour work provides useful calibration for how these models generalise. A DL MSI predictor developed and validated on prostate cancer whole-slide images achieved an AUROC of 0.78 on internal validation and 0.72 on external temporal validation [121]. Both figures fall below CRC-specific performance, which is consistent with MSI-associated morphology being more pronounced in colorectal tissue, and serves as a reminder that performance does not travel between tumour types without revalidation.

### 4.4. Multi-Target and Multimodal Prediction, Explainability and Deployment

Transformer architectures have displaced convolutional networks as the state of the art for biomarker prediction from CRC histology. Across a large multicentric cohort, a fully transformer-based pipeline combining a pretrained encoder with a transformer patch-aggregation network improved performance, generalisability, data efficiency and interpretability relative to CNN-based approaches [122]. Of these the gain in data efficiency is the most consequential, since it lowers the cohort size at which a new biomarker target becomes trainable. Multi-target transformer models extend this by predicting numerous genetic alterations and associated phenotypes simultaneously. In a multicentre cohort study, such models predicted biomarkers from pathology slides at performance matching and partly exceeding single-target transformers, with a mean AUROC of 0.78 for *BRAF*, 0.88 for hypermutation, 0.93 for MSI and 0.86 for *RNF43* [25]. Multi-target approaches offer improved efficiency and, in several settings, improved predictive power relative to training one model per biomarker.

Integrating image features with nuclear segmentation features using multimodal compact bilinear pooling increased the AUROC by 1–3% and the recall by 5–11% relative to models relying on image features alone, with external validation achieving an AUROC of 0.81 and a recall of 0.80 for MSI prediction [123]. Knowledge distillation has enabled lightweight models that maintain diagnostic accuracy while reducing computational requirements, achieving accuracy above 93% with an AUROC above 0.91 [124]. Deep Gaussian process approaches add calibrated uncertainty estimation to MSI and immunotherapy response prediction from histology [26]. Clinically, this is no small thing: a model able to decline prediction on ambiguous slides is safer than one obliged to answer.

Modality fusion extends this further. A multimodal DL model integrating preoperative contrast-enhanced CT with postoperative whole-slide images achieved an AUROC of 0.905 on external test sets for MSI prediction, outperforming single-modality alternatives and demonstrating generalisability across centres [125]. A CT-based model integrating DL features, handcrafted radiomics and body composition achieved an AUROC of 0.882 in training and 0.768–0.803 in external validation cohorts, with decision curve analysis indicating clinical benefit [126]. Ensemble learning integrating pretreatment colonoscopy images with routine clinical data achieved an AUROC of 0.896 for non-invasive MSI prediction [127].

Classical ML retains a competitive edge over DL in settings with limited data or where interpretability of model decisions is required, and the choice between them should follow the deployment constraint, not novelty. SHAP analysis and Grad-CAM have become standard tools for interpretability in this domain. Applied to MSI prediction, they identify debris, lymphocytes and necrotic cells as the morphological features driving model output [27]. These are biologically coherent with the known immunogenic phenotype of MSI-H tumours, so the explanation supports the prediction and does not merely describe it. Interpretable ensemble approaches employing Grad-CAM for image models and SHAP for clinical models enhance clinical acceptance and support deployment in routine diagnostic workflows [127].

### 4.5. Pathomics and Tumour Microenvironment Assessment

Computational pathomics, which applies feature extraction and ML to whole-slide histopathology, has become a powerful approach to biomarker discovery and prognosis prediction. Its prognostic potential was demonstrated at scale in a discovery and validation study across four cohorts, where a DL biomarker derived from conventional slides stratified patients by cancer-specific survival independently of established clinicopathological markers, with a hazard ratio of 3.84 for poor against good prognosis after adjustment [128]. Digitisation of slides and application of DL enables automated identification of tumour-infiltrating lymphocytes, quantification of tumour–stroma ratio, assessment of tumour budding and identification of necrotic regions, all associated with prognosis and treatment response.

A pathomics-based ML model for predicting pathological complete response in locally advanced rectal cancer achieved an AUROC of 1.000 on an internal dataset and 0.950 on an external dataset [129]. A perfect internal figure is best read as evidence of overfitting to the development cohort; the external result is the more informative number. Pathomic signatures derived from haematoxylin and eosin-stained whole-slide images have identified tumour cell infiltration, adipocyte accumulation, fibrous tissue deposition and stromal infiltration as independent predictors of overall and disease-free survival and of chemotherapy benefit.

Imaging can also characterise the immune microenvironment non-invasively. CT-based DL radiomics achieved an AUROC of 0.892 for tumour–stroma ratio prediction and 0.772 for tumour immune microenvironment score classification, with decision curve analysis confirming clinical utility across threshold probabilities [130]. Pathomic features are often more biologically interpretable than radiomic ones. Attention visualisation has, for instance, reinforced the prognostic weight of tumour budding, the isolated cancer cells or small clusters invading the tumour margin, relative to conventionally assessed invasion patterns.

### 4.6. Treatment Response Prediction

ML-based radiomics models predicting treatment response in locally advanced rectal cancer have achieved promising performance, with MRI radiomic features obtaining the best predictive accuracy of 0.88 among modalities compared [131]. For colorectal liver metastases, PET/CT radiomics signatures predicted immunotherapy response with an AUROC of 0.784 in external validation [132]. Baseline-referenced delta radiomics, which incorporates the relationship between radiomic features and tumour size over time, achieved a sensitivity, a specificity and a balanced accuracy of 66–97%, 81–97% and 80–90%, respectively, for predicting chemotherapy response in liver metastases [133].

In the neoadjuvant setting, the SRADL model integrating deep learning with sub-regional radiomic signatures predicted pathological complete response in locally advanced rectal cancer with an AUROC of 0.925 in training and 0.902–0.915 in external test cohorts, and patients in the predicted complete response group had longer survival across all cohorts [134]. MRI-based radiomics from intratumoural and peritumoural regions discriminated treatment response with AUROCs of 0.797–0.799 in training and validation datasets, with a combined model integrating tumour size, MRI-detected extramural venous invasion and a support vector machine-derived radiomic signature validated by decision curve analysis in a large multicentre study [135].

## 5. Machine Learning for Prognosis and Risk Stratification

ML algorithms have demonstrated potential for predicting survival outcomes in CRC. A multicentre national study analysing 1853 patients found that time from diagnosis, age, tumour size, metastatic status, lymph node involvement and treatment type were the most influential predictors by mean decrease in Gini criterion, with the best-performing models reaching an AUROC of approximately 0.70 for mortality classification [136]. A separate model for five-year survival prediction found XGBoost gave the best combination of discrimination and generalisability, with an AUROC of 0.906 on internal validation and 0.813 on external validation [137].

The gap between these two studies is instructive. Both are ML survival models in CRC, yet they differ in cohort, feature set, and outcome definition, and their performance differs accordingly. Reported AUROC values cannot be compared across studies without attention to what was predicted, in whom it was predicted and against which validation set. This caveat applies to every performance figure summarised in Table 1. Read across rather than down, however, the table shows a pattern the individual studies cannot. The highest values cluster on tasks where the target is a molecular or pathological state already physically encoded in the input, such as MSI inferred from histology or pathological response inferred from post-treatment imaging. There, the AUROC commonly falls between 0.90 and 0.98. Prediction of future clinical events sits considerably lower, generally between 0.70 and 0.85. The distinction is between recognising something already present in the sample and forecasting something that has not yet happened, and it is the second of these that adjuvant decisions actually require. A second regularity is equally consistent: wherever both internal and external figures are reported, performance falls, from 0.906 to 0.813 for five-year survival, from 0.872 to 0.718 for recurrence, and from 1.000 to 0.950 for pathological complete response. Single-cohort discrimination should therefore be treated as an upper bound rather than an estimate.

Risk prediction for recurrence is central to postoperative treatment decisions. ML applied to standardised pathology reports for stage II and III CRC achieved an accuracy of 0.87 with a specificity of 1.0 in testing, with age and lymph node ratio selected as important prognostic factors by both multivariable analysis and ML [138]. A tertiary lymphoid structure-based ML model for recurrence prediction found LightGBM gave the most balanced performance, with an AUROC of 0.872 in training and 0.718 in internal validation; SHAP analysis ranked the invasive-front tertiary lymphoid structure score as the most influential predictor, and the model separated high- and low-risk groups with significantly different disease-free survival [139].

An interpretable CT-based model integrating imaging with clinicopathological factors for three-year disease-free survival in stage II CRC achieved an AUROC of 0.811 and 0.846 in test and validation cohorts, with Kaplan–Meier curves confirming effective stratification and SHAP identifying high Radscore, irregular intestinal outer edge and advanced age as key risk factors [140].

ML models predicting metastasis using demographic, lifestyle, nutritional and clinical factors have achieved encouraging results, with LightGBM reaching an 88.14% accuracy and an AUROC of 0.90; history of inflammatory bowel disease, family history of CRC, number of involved lymph nodes, fruit intake and tumour size were identified as important predictors [141]. A systematic review and meta-analysis of ML-based prediction models for early-onset CRC risk found that model performance varied considerably with the predictor set and validation approach, which indicates that risk model development in this area has outpaced rigorous external validation [142].

## 6. Clinical Applications: MSI-H, Immunotherapy and Treatment Selection

### 6.1. Microsatellite Instability and Immunotherapy Efficacy

MSI-H status, resulting from deficient mismatch repair, is arguably the single most clinically impactful precision oncology biomarker in CRC, identifying patients with exceptional immunotherapy benefit [143]. Its arrival has restructured CRC treatment more broadly, moving immunotherapy from a salvage option to a first-line consideration within a defined molecular subgroup and prompting reassessment of where checkpoint blockade sits relative to cytotoxic and targeted agents across the disease course [144]. MSI-H CRC carries a profound mutational burden, typically 10–15 mutations/Mb against under 1 mutation/Mb in microsatellite-stable (MSS) tumours, and generates numerous neoantigens that activate anti-tumour immune responses [12,145]. This heightened immunogenicity renders MSI-H tumours sensitive to immune checkpoint inhibitors: a meta-analysis of 13 randomised trials including 1633 MSI-H patients found that immunotherapy reduced the risk of death by 65% compared with chemotherapy (HR: 0.35; 95% CI: 0.27–0.46) [145].

Regulatory approval of pembrolizumab for MSI-H/dMMR solid tumours established MSI-H as a tissue-agnostic biomarker for immunotherapy eligibility [143]. The observation underlying this now rests on a phase 2 study showing that mismatch repair status predicted benefit from programmed cell death protein 1 (PD-1) blockade across tumour types, with immune-related objective response in 40% of dMMR colorectal cancers against none in mismatch repair-proficient disease [146]. The pivotal randomised evidence in CRC then came from KEYNOTE-177, the first phase III trial comparing PD-1 blockade with standard chemotherapy as the first-line treatment of MSI-H/dMMR metastatic disease, which more than doubled median progression-free survival (16.5 versus 8.2 months) and established pembrolizumab as the first-line standard of care [147]. Durable benefit has already been shown in pre-treated disease by CheckMate 142, in which nivolumab produced investigator-assessed objective response in 31.1% of previously treated dMMR/MSI-H patients with disease control for twelve weeks or longer in 69% [148]. Across these trials, response rates exceeded 50% in the first-line setting, with many patients experiencing durable complete responses. Ten-year follow-up of the phase 2 pembrolizumab trial in MSI-H/dMMR advanced solid tumours demonstrates sustained benefit in a substantial subset, suggesting potential for cure in some patients [149].

A network meta-analysis comparing pembrolizumab, nivolumab and nivolumab plus ipilimumab found the combination showed the highest efficacy across endpoints: objective response rate: 0.54, overall survival HR: 0.84, progression-free survival HR: 0.73, and disease control rate: 0.82 [150]. Real-world outcomes from community hospitals validated these findings, with a 72.7% overall response and an 81.8% disease control in MSI-H patients receiving combination, sequential and single-agent regimens [151]. Cost-effectiveness analysis found dual immunotherapy with nivolumab plus ipilimumab economically favourable for MSI-H/dMMR advanced CRC in the United States healthcare setting [152].

A substantial minority of MSI-H patients still show primary resistance or early progression. Biomarkers are therefore needed within the MSI-H group, to separate those who benefit from monotherapy from those who require combination approaches. TMB, while correlated with MSI-H status, shows variable association with response within the MSI-H population; genomic landscape analyses stratifying dMMR/MSI-H gastrointestinal cancers by TMB have begun to characterise this heterogeneity [153]. Inactivating mutations in *beta-2 microglobulin* (*B2M*), *JAK1*, *JAK2* or *PTEN* associate with immunotherapy resistance even in the MSI-H context, supporting comprehensive genomic profiling beyond MSI status alone [154].

### 6.2. Beyond MSI-H: Predictive Biomarkers, Resistance and Microsatellite-Stable Disease

Integration of TMB with additional biomarkers improves prediction of immunotherapy response, with multi-biomarker models combining TMB, programmed death-ligand 1 (PD-L1) and immune infiltration outperforming any single biomarker [155]. Combining TMB with MSI status, *POLE*/*POLD1* mutations and immune checkpoint molecule expression creates a panel capturing multiple resistance mechanisms [156,157].

CDX-2 expression has emerged as a prognostic-predictive marker in MSI-H mCRC treated with immunotherapy, with CDX-2-positive patients showing 81% twelve-month progression-free survival against 0% in CDX-2-negative patients (*p* = 0.0011) [158]. Such a stark separation requires confirmation in larger prospective cohorts before it can guide practice.

Specific mismatch repair protein loss patterns may also predict response. MutS complex loss (MSH2/MSH6 co-loss) was associated with better immunotherapy outcomes than MutL complex loss (MLH1/PMS2 co-loss) in gastrointestinal non-colorectal cancers, suggesting refined MMR subtyping could further personalise treatment selection [159].

Approximately 95% of CRC is microsatellite stable, and immune checkpoint inhibitors alone have shown limited efficacy in this population. Strategies combining immunotherapy with chemotherapy, targeted therapy or radiotherapy are under active investigation [144,160]. A comprehensive review of approaches to overcome immunotherapy resistance in MSS CRC identified several promising combination strategies and emphasised the importance of immune microenvironment assessment in guiding therapeutic decisions [161]. Here, non-invasive characterisation of the microenvironment, whether by CT-based deep learning radiomics [130] or pathomic quantification of immune infiltration, has the greatest potential to contribute. Its role would be to identify the MSS subgroup with an inflamed phenotype, the group most plausibly amenable to combination immunotherapy.

Computational approaches are beginning to address this population directly, and with a different logic from the biomarker prediction described in Section 4. Rather than inferring a molecular label, one machine learning scoring system quantifies how closely a microsatellite-stable tumour resembles the MSI-H immune phenotype, combining tumour-infiltrating lymphocyte features, mutational profile and immune microenvironment composition to identify the minority of MSS tumours with high immunoactivity that might plausibly respond to checkpoint blockade [162]. The target here is not a molecular state with an established reference assay but a latent phenotype defined by resemblance to a responsive group, which makes validation harder: There is no ground truth against which to score the prediction other than response itself. The burden therefore falls on prospective evaluation rather than on retrospective discrimination, and this remains the principal obstacle to cracking the MSS immunotherapy barrier computationally.

### 6.3. Neoadjuvant and Adjuvant Immunotherapy

Success in metastatic MSI-H disease has driven investigation of earlier treatment settings. Multiple phase II trials have reported pathological complete response (pCR) rates with neoadjuvant immunotherapy in MSI-H locally advanced CRC ranging from 47% to 73%, depending on population and regimen [163]. Radiological and pathological response diverge substantially and predominantly in the direction of imaging underestimating response: the overall discordance reaches 59.6%, and in one series, only 2.7% of patients achieving pathological complete response showed radiological complete remission [164]. The risk in this setting is therefore not that imaging misses residual tumour but that it reports residual disease in patients who have already achieved a complete pathological response, so that organ preservation decisions cannot rest on imaging alone.

The most arresting result in this setting came from a prospective phase 2 study of single-agent dostarlimab, given every three weeks for six months in dMMR stage II–III rectal adenocarcinoma and intended to be followed by chemoradiotherapy and surgery. All 12 patients completing treatment achieved a clinical complete response on MRI, FDG-PET, endoscopy, digital rectal examination and biopsy, and none required chemoradiotherapy or surgery [165]. Several qualifications apply: the cohort is small and single-centre, and clinical complete response is not the same endpoint as pathological complete response, and no specimen was available for pathological confirmation. The direction of discordance described above [164] makes an imaging-based complete response more likely to be genuine than to conceal residual tumour, but durable follow-up remains the only way to establish that. Even so, the finding converts organ preservation from an aspiration into a testable primary strategy for this molecular subgroup. Real-world data from a multicentre French AGEO study of dMMR/MSI non-metastatic colon cancer receiving neoadjuvant immunotherapy reported pCR in 42% and major pathological response in 64%, with grade ≥ 3 toxicity in 19% including one potential toxic death [166]. An interim analysis of the IMHOTEP trial of perioperative pembrolizumab in 70 patients with localised dMMR/MSI tumours reported a pCR rate of 38.9%, with differential response by tumour type (40.7% in CRC, 25.0% in gastro-oesophageal cancer, 0% in endometrial cancer) [167]. A retrospective case series evaluating prolgolimab in 26 MSI/dMMR patients with neoadjuvant intent reported 100% objective response, 40% complete clinical response, and pCR in 7 of 9 operated patients [168]. The small sample and retrospective design temper the apparent magnitude of these figures.

Adjuvant immunotherapy for high-risk stage II–III MSI-H CRC represents an emerging opportunity, with long-term survival data from neoadjuvant and adjuvant ICI use in dMMR/MSI-H colorectal and gastric cancers now available [169]. Selection criteria remain incompletely defined: which patients genuinely require adjuvant therapy, and which carry a recurrence risk low enough to justify observation. Integration of ctDNA clearance kinetics, immune infiltration profiling and multi-omic signatures may enable more precise identification of MSI-H patients most likely to benefit from intensification [88,157].

## 7. Integration, Implementation and Future Perspectives

### 7.1. Clinical Translation, Standardisation and Regulatory Considerations

Implementing next-generation biomarkers and AI models in routine CRC care requires overcoming organisational, economic and regulatory challenges. Standardisation of biomarker testing platforms, assay quality metrics and interpretation guidelines remains incomplete. Different NGS panels, liquid biopsy assays and radiomics software yield variable results, which complicates multi-institutional collaboration and biomarker-driven trial design [106,111]. Initiatives seeking consensus standards for liquid biopsy testing are underway, but substantial work remains to harmonise methodologies globally.

Cost operates as an implicit constraint throughout this review and warrants explicit treatment, because the economic case differs sharply between the two dominant strategies. For molecular profiling, wider panels do not necessarily buy better decisions. A Markov model comparing genomic tests in stage II disease found the Immunoscore assay dominant at a willingness-to-pay threshold of US$50,000 per quality-adjusted life year, at US$23,564 for 3.903 QALYs against US$24,545 for a 12-gene assay, US$28,374 for an 18-gene assay and US$33,315 for a 482-gene signature [170]. Deep learning applied to haematoxylin and eosin-stained slides occupies a different economic position entirely: the marginal cost of inference on a slide already produced for diagnosis approaches zero, with expenditure concentrated in scanner capital, storage and validation rather than in per-test consumables. That asymmetry bears directly on the equity argument of Section 7.2, since a method whose cost is dominated by one-off infrastructure is more tractable in a resource-constrained setting than one whose cost recurs with every patient. The same logic governs screening at the population scale, where United States expenditure on colorectal cancer screening reached US$27.5 billion in 2021 and colonoscopy accounted for 54.8% of it while representing only 9.3% of initial tests [171].

Ensemble and multi-omic integration frameworks are approaching the performance thresholds needed for clinical use. A unified ML framework incorporating unmatched exomic and transcriptomic data classified immunotherapy response in non-small cell lung cancer with an AUROC of 0.80 through ensemble approaches integrating TMB, CXCL9 expression, PD-L1, line of therapy and dual immunotherapy status [31]; multi-omic explainable ML has similarly improved treatment outcome prediction across cancer types [32]. Integrated ML and genetic algorithm-driven multi-omic analysis has been applied to pan-cancer immunotherapy response prediction, with the CRC subset showing performance consistent with the pan-cancer result [30].

**Figure 2:**
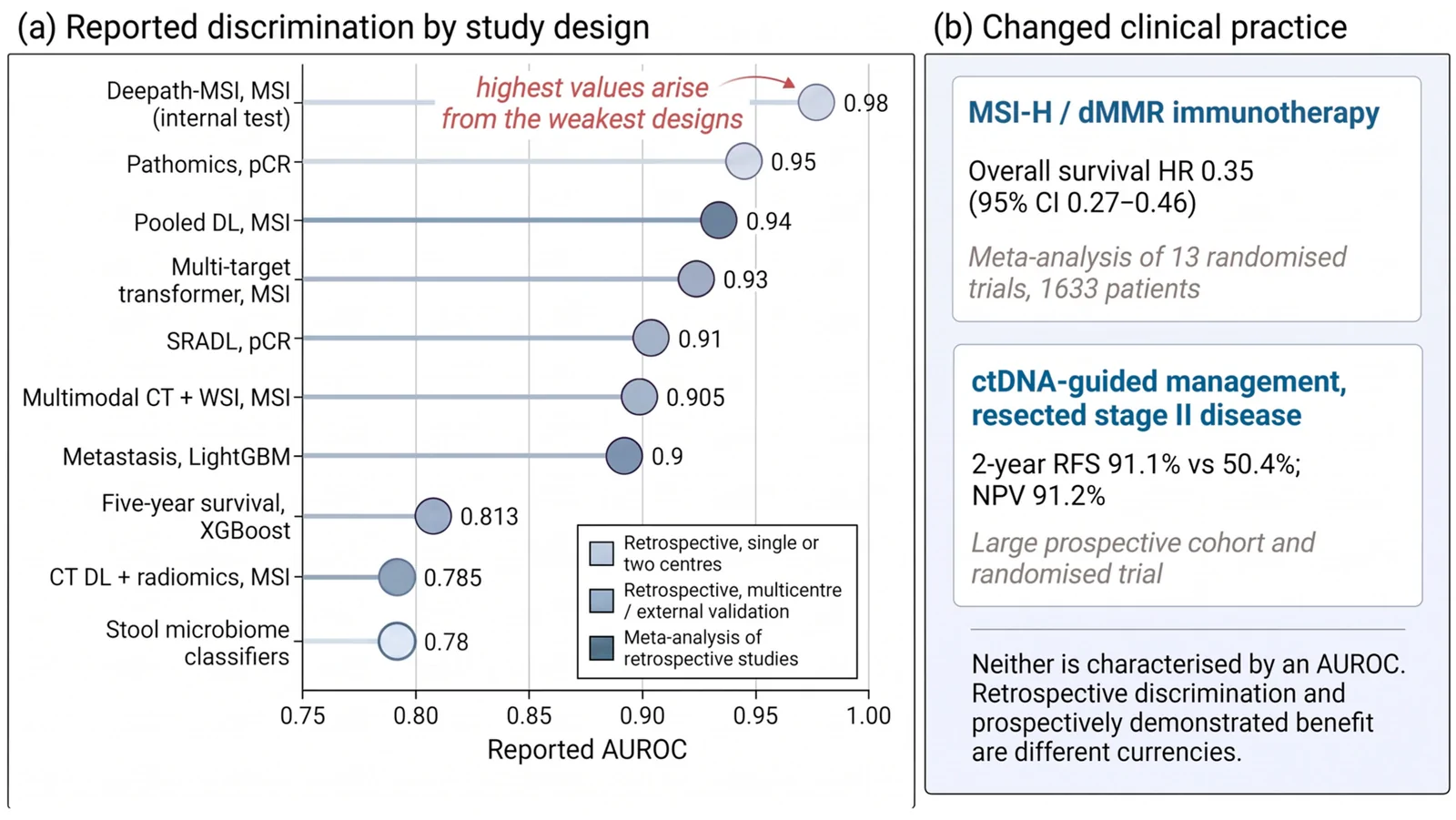
Reported discrimination against strength of study design. (**a**) AUROC values from Table 1, ordered by magnitude and shaded by design; the studies shown are, in descending order of reported AUROC, Feng et al. [120], Zhang et al. [129], Wang et al. [119], Gustav et al. [25], Wu et al. [134], Tang et al. [125], Nopour [141], Nopour [137], Han et al. [126] and the stool microbiome classifiers of references [53,54,55,56,57,58,59,60,61,62,63,64]. The two highest values arise from single- or two-centre retrospective series; across these studies, discrimination and design strength are not correlated (*r* = 0.02), so higher reported performance does not indicate stronger evidence. (**b**) The two applications that have changed clinical practice, immune checkpoint inhibition in MSI-H disease [145] and ctDNA-guided management of resected stage II disease [89,97], report no discrimination metric, being characterised instead by randomised outcome measures.

Two features of Table 2 are worth drawing out, because neither is visible from any single row. The first is how few applications rest on randomised or large prospective evidence. Only ctDNA-guided management of resected stage II disease and immune checkpoint inhibition in MSI-H tumours meet that standard; everything else rests on meta-analyses of retrospective series, multicentre external validation at best or single-centre cohorts. The second is that higher reported discrimination does not indicate stronger evidence. Across the studies in Table 1, the two are uncorrelated (*r* = 0.02), and the highest values, from deep learning on histopathology and from pathomic prediction of treatment response, arise from single- or two-centre retrospective series (Figure 2a). The two applications that have actually changed practice, by contrast, are described not by an AUROC but by a hazard ratio and a negative predictive value obtained under randomisation. Discrimination measured retrospectively and benefit demonstrated prospectively are different currencies, and the field currently holds far more of the former (Figure 2b).

Regulatory pathways for clinical AI validation require clarification. Frameworks designed for traditional drugs and static devices accommodate poorly those models that evolve through retraining on new data. Several agencies are developing pathways that recognise how fixed models degrade as practice and populations shift. Implementing iteratively updated systems that improve over time while retaining safety, auditability and interpretability will require collaboration among developers, clinicians, regulators and patients. Unresolved issues in ethical governance and data stewardship sit alongside the technical problems of harmonisation and generalisability [111].

For liquid biopsy specifically, several current issues in NGS-based ctDNA analysis remain barriers to interoperability between centres: pre-analytical variability, panel composition, variant calling thresholds and reporting conventions [106].

### 7.2. Data Equity, Global Implementation and Prospective Validation

Most AI models and multi-omic datasets are derived from populations of European ancestry, which may limit generalisability to groups whose genetic architecture, environmental exposures, and treatment responses differ. Efforts to expand cohort diversity, validate models across ancestry groups and establish equity-focused implementation strategies remain urgently needed. Real-world ctDNA profiling across Asia and the Middle East illustrates both the value and the feasibility of broadening the geographical base of genomic evidence [103].

Ensuring that precision medicine benefits under-resourced populations, rather than widening disparities, requires deliberate policy: open models, affordable testing and capacity building. Reviews of cancer screening and early diagnosis in resource-constrained settings emphasise that liquid biopsy innovation and AI-driven imaging must be coupled with strategic adoption, scalable low-resource methods and strengthened primary healthcare infrastructure to deliver equitable benefit [172].

Multiple prospective randomised trials are underway to validate ctDNA-guided treatment decisions and AI-driven prognostic models in CRC. These test whether ctDNA-informed escalation or de-escalation, compared with conventional clinicopathological risk stratification, improves outcomes while reducing unnecessary toxicity [8,85]. For de-escalation in stage II colon cancer, the question has now been answered affirmatively with mature five-year data [97,98]; results from CIRCULATE-JAPAN and comparable international collaborations will determine whether the same holds for escalation and for other stages. The equivalent standard for AI models is prospective, multicentre, pre-registered evaluation against a defined clinical endpoint. Very few of the models summarised in this review have met it. This gap between retrospective discrimination and demonstrated clinical benefit is the central obstacle to adoption.

### 7.3. Emerging Technologies

Several directions will shape the next phase. Integrating radiomics with genomics and longitudinal data supports patient-specific digital twin systems [111]; federated learning enables multi-institutional training without centralising patient data, addressing privacy and cohort diversity together; and spatial transcriptomics and single-cell approaches promise finer characterisation of heterogeneity than bulk profiling allows, with integration with pathomic features representing a natural next step since both describe the same spatial substrate [10,19].

Novel modalities should not, however, crowd out established but under-integrated ones. Genomic instability generates the molecular heterogeneity the rest of this review attempts to measure, yet the direct indices of that process appear in almost none of the integrative models catalogued above. Telomere length and telomerase activity covary systematically with tumour site, MSI status, histology and stage [1,75], and micronucleus frequency offers a cytogenetic readout of chromosomal instability that is prospectively measurable in peripheral blood, inexpensive relative to sequencing and serially samplable across a treatment course [77,78]. Neither has been combined with molecular, pathomic or radiomic features in any published integrative model for colorectal cancer.

The combination is worth testing on three grounds. The information is plausibly non-redundant, since instability indices measure a mutational process whereas the signatures of Section 2.5 measure its products. The error structures differ, so a rule-in marker complements ctDNA-based detection, whose value rests on the opposite profile (Section 3.2). In addition, these features are cheap, blood-based and free of sequencing infrastructure, which bears on the equity considerations of Section 7.2.

The obstacle is measurement rather than modelling, the same obstacle identified in Section 2.7. Progress depends on assay harmonisation preceding model development, inverting the sequence followed by most work reviewed here, in which model performance has been optimised on measurements whose reproducibility was never established.

## 8. Conclusions

Next-generation biomarkers and artificial intelligence together mark a substantive shift in colorectal cancer management, supporting earlier detection, more precise molecular characterisation, individualised treatment selection and dynamic disease monitoring. Multi-omic technologies across genomics, transcriptomics, proteomics, metabolomics and microbiomics now permit comprehensive tumour profiling, and deep learning has reached clinical-grade performance for predicting molecular biomarkers, MSI above all, directly from routine histopathology slides.

Liquid biopsy, and ctDNA-based assays in particular, have become a powerful tool for minimal residual disease detection and monitoring of treatment response; its high negative predictive values support a role in adjuvant therapy decisions. Randomised evidence now extends this further, showing that ctDNA-guided management can safely reduce adjuvant chemotherapy use in stage II colon cancer without compromising survival. ML has achieved useful performance in predicting survival, recurrence, metastasis and treatment response. At the same time, the CMS framework continues to provide a basis for stratification informing both prognosis and therapy selection. Of all the markers considered here, MSI-H status remains the clearest instance of one that has changed practice outright.

Difficulties in standardisation, validation and implementation nevertheless remain, and they are more often analytical than biological. The performance figures assembled throughout this review are derived predominantly from retrospective cohorts with heterogeneous endpoints and inconsistent external validation, and the highest reported accuracies are frequently the least externally confirmed (Table 1 and Table 2). Telomere biology illustrates how durable this constraint can be. Three decades of secure mechanistic evidence have not produced a clinically actionable colorectal biomarker, and the reason is not that the underlying observation failed to replicate; it is that the assays resist standardisation (Section 2.7). There is little reason to expect computational sophistication to clear a bar that measurement reproducibility has not. Translation into routine practice will require assay harmonisation, computational infrastructure, regulatory pathways suited to adaptive models, demonstrated cost-effectiveness and validation across ancestrally and geographically diverse populations. Future research should prioritise prospective clinical validation, principled multi-omic integration strategies and explainable frameworks that support clinician trust and shared decision-making.

Combining molecular biomarkers, imaging features and clinical data within rigorous computational frameworks holds real promise for moving CRC management away from population-level protocols and toward individualised care. Whether that promise is realised will depend less on further gains in retrospective discrimination than on the discipline of prospective validation and on equitable implementation.

## Figures and Tables

**Table 1:** Reported performance of machine learning approaches across clinical prediction tasks in colorectal cancer. Values are as reported in the cited studies and are not directly comparable across rows, since cohorts, feature sets, outcome definitions and validation strategies differ.

Prediction Task	Approach	Setting and Cohort	Reported Performance	Ref.
MSI status from histopathology	Pooled DL (30 studies)	Meta-analysis; 30 studies	AUROC: 0.94; sens.: 0.90; spec.: 0.86	[119]
MSI status from histopathology	Feature-based MIL (Deepath-MSI)	Internal test set; 5070 slides	AUROC: 0.98; 92% accuracy	[120]
MSI, *BRAF*, hypermutation, *RNF43*	Multi-target transformer	Multicentre cohort; 1912 patients	AUROC: 0.93/0.78/0.88/0.86	[25]
MSI status	Multimodal DL (CT + whole-slide)	External test; 305 patients, 3 centres	AUROC: 0.905	[125]
MSI status	CT DL + radiomics + body composition	External validation; 873 patients, 3 centres	AUROC: 0.768–0.803	[126]
Five-year survival	XGBoost	External validation; 1062 cases	AUROC: 0.813 (internal: 0.906)	[137]
Mortality classification	Multiple algorithms	Multicentre national cohort; 1853 patients	AUROC: ≈0.70	[136]
Recurrence (stages II–III)	Logistic regression on pathology reports	Internal test set; 1073 patients	Accuracy: 0.87; spec.: 1.0	[138]
Recurrence (TLS-based)	LightGBM	Internal validation; 224 + 89 patients	AUROC: 0.718 (training: 0.872)	[139]
Three-year disease-free survival	Interpretable CT-based model	Test/validation; 769 patients	AUROC: 0.811/0.846	[140]
Metastasis	LightGBM	External validation; 1156 + 115 patients	AUROC: 0.90; accuracy: 88.1%	[141]
pCR after neoadjuvant CRT	Sub-regional radiomics + DL (SRADL)	External test; 768 patients, 3 hospitals	AUROC: 0.902–0.915	[134]
pCR after neoadjuvant CRT	Pathomics ML	External dataset; 294 patients, two institutions	AUROC: 0.950 (internal: 1.000)	[129]

**Table 2:** Clinical applications of next-generation biomarkers and AI in colorectal cancer, with current evidence status.

Application	Biomarker/Method	Reported Performance	Clinical Utility	Evidence Status
MRD detection after resection	Tissue-free ctDNA (genomic + methylation)	NPV: 91.2%; RFS: 91.1% vs. 50.4% (*n* = 214)	Adjuvant therapy decisions	Large prospective cohort [89]
Recurrence risk, stage II	Postoperative ctDNA	Pooled RR: 3.66 (meta-analysis of fewer than 10 studies)	Escalation vs. observation	Systematic review/meta-analysis [91]
MSI status	DL on routine histopathology	Pooled AUROC: 0.94 (30 studies)	Immunotherapy triage	Meta-analysis, external validation [25,119]
MSI status, non-invasive	CT/colonoscopy-based models	AUROC: 0.77–0.91 (incl. *n* = 1844)	Pre-screening before confirmatory testing	Multicentre external validation [125,126,127]
Immunotherapy benefit	MSI-H/dMMR	OS HR: 0.35 vs. chemotherapy (13 trials, *n* = 1633)	Treatment selection	Meta-analysis of randomised trials [145]
Prognostic stratification	CMS classification	Significant OS differences by subtype (4151 patients, 18 studies)	Risk stratification, trial design	Established; adoption limited by assay access [39,40]
Neoadjuvant response	Radiomics/pathomics	AUROC: 0.79–0.92 (incl. *n* = 768)	Organ preservation decisions	Multicentre validation [129,134,135]
Early detection	Stool microbiome ML classifiers	AUROC: 0.61–0.98 (internal); 0.70–0.87 (external); largest single pooled analysis: 3741 metagenomes from 18 cohorts	Non-invasive screening adjunct	Predominantly retrospective case-control; prospective screening evaluation lacking [53,54,55,56,57,58,59,60,61,62,63,64]
Early detection	Blood-based methylation markers	Detectable in plasma early in carcinogenesis	Population screening adjunct	Assay standardisation incomplete; trials ongoing [38]
Prognostic stratification	Telomerase activity/*TERT* expression (tissue)	Detected in 81–90% of CRC; absent in normal colon	Not yet actionable; candidate adjunct to staging	Consistent tissue-level findings; blood assay specificity and standardisation unresolved [1,76]
Treatment response monitoring	Micronucleus frequency (peripheral blood lymphocytes)	Spec.: 87.0%, sens.: 36% for progressive disease (*n* = 55 + 21)	Rule-in adjunct during systemic therapy	Prospective single-centre cohort; needing external validation [77]

## Data Availability

No new data were created or analysed in this study. Data sharing does not apply to this article.

## Abbreviations

The following abbreviations are used in this manuscript:

AI	artificial intelligence
AUROC	area under the receiver operating characteristic curve
B2M	beta-2 microglobulin
CEA	carcinoembryonic antigen
CMS	consensus molecular subtype
CRC	colorectal cancer
CRT	chemoradiotherapy
CT	computed tomography
ctDNA	circulating tumour DNA
DL	deep learning
dMMR	mismatch repair deficient
EGFR	epidermal growth factor receptor
Grad-CAM	gradient-weighted class activation mapping
HER2	human epidermal growth factor receptor 2
ICI	immune checkpoint inhibitor
MIL	multiple-instance learning
ML	machine learning
MRD	minimal residual disease
MRI	magnetic resonance imaging
MSI	microsatellite instability
MSI-H	microsatellite instability-high
MSS	microsatellite stable
NET	neutrophil extracellular trap
NGS	next-generation sequencing
NPV	negative predictive value
PCR	polymerase chain reaction
pCR	pathological complete response
PD-1	programmed cell death protein 1
PD-L1	programmed death-ligand 1
PET	positron emission tomography
SHAP	SHapley Additive exPlanations
TERT	telomerase reverse transcriptase
TLS	tertiary lymphoid structure
TMB	tumour mutational burden
VAF	variant allele frequency
XAI	explainable artificial intelligence
